# Evaluation of the performance of three tenodesis techniques for the treatment of scapholunate instability: flexion-extension and radial-ulnar deviation

**DOI:** 10.1007/s11517-017-1748-1

**Published:** 2017-11-25

**Authors:** Teresa Alonso-Rasgado, Qing-Hang Zhang, David Jimenez-Cruz, Colin Bailey, Elizabeth Pinder, Avanthi Mandaleson, Sumedh Talwalkar

**Affiliations:** 1 Bioengineering Research Group, School of Materials, University of Manchester, Manchester, M13 9PL UK; 2 Queen Mary, University of London, London, UK; 3 Wrightington Hospital, Wigan and Leigh NHS Foundation Trust, Lancashire, UK

**Keywords:** Finite element, Scapholunate instability, Reconstruction, Scapholunate gap, Scapholunate angle

## Abstract

Chronic scapholunate ligament (SL) injuries are difficult to treat and can lead to wrist dysfunction. Whilst several tendon reconstruction techniques have been employed in the management of SL instability, SL gap reappearance after surgery has been reported. Using a finite element model and cadaveric study data, we investigated the performance of the Corella, scapholunate axis (SLAM) and modified Brunelli tenodesis (MBT) techniques. Scapholunate dorsal and volar gap and angle were obtained following virtual surgery undertaken using each of the three reconstruction methods with the wrist positioned in flexion, extension, ulnar deviation and radial deviation, in addition to the ulnar-deviated clenched fist and neutral positions. From the study, it was found that, following simulated scapholunate interosseous ligament rupture, the Corella technique was better able to restore the SL gap and angle close to the intact ligament for all wrist positions investigated, followed by SLAM and MBT. The results suggest that for the tendon reconstruction techniques, the use of multiple junction points between scaphoid and lunate may be of benefit.

**Graphical abstract:**
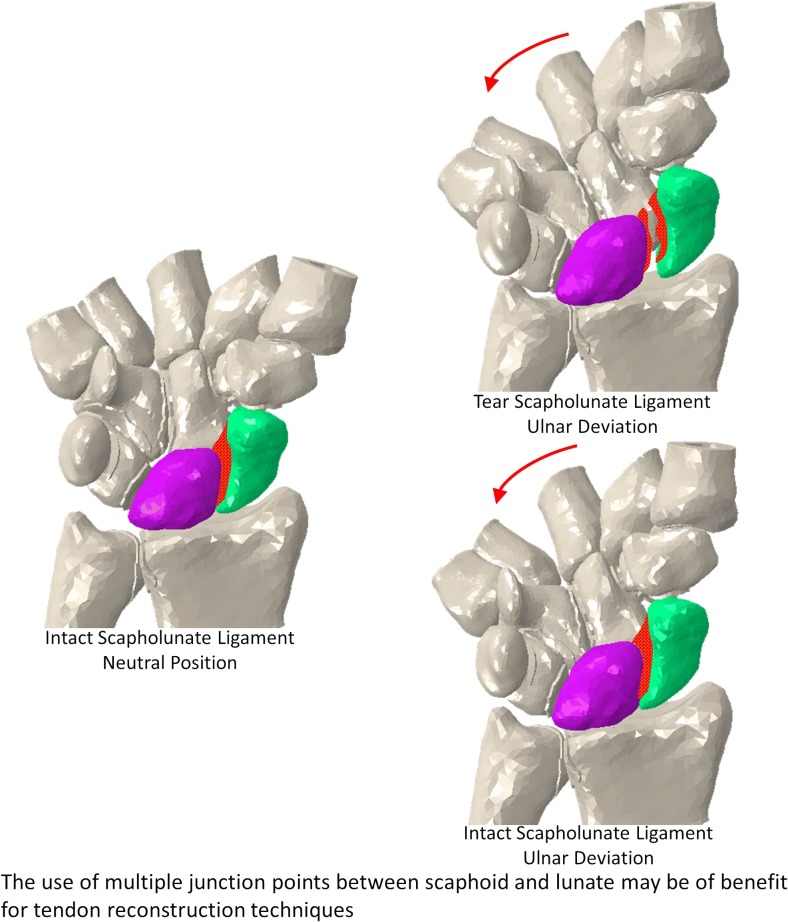
The use of multiple junction points between scaphoid and lunate may be of benefit for tendon reconstruction techniques.

The use of multiple junction points between scaphoid and lunate may be of benefit for tendon reconstruction techniques.

## Introduction

Scapholunate interosseous ligament (SLIL) injury is a relatively common [1, 2] wrist ligament condition which if not treated successfully may lead to carpal instability and degenerative osteoarthrosis [3]. SLIL injury occurs most frequently with the wrist positioned in extension, ulnar deviation and carpal supination. Treatment of scapholunate instability depends upon the severity of the injury which can vary widely [4]. For subjects presenting with dynamic scapholunate or static reducible instabilities, ligamentous reconstruction is a consideration [5].

In flexion-extension of the wrist, the lunate rotates over the radius in the dorsal direction during flexion and in the volar direction during extension [6]; therefore, reduction and stabilisation of the dorsal gap is important in flexion, whereas for extension the volar gap is significant. Until recently, SLIL reconstruction techniques, including the Brunelli tenodesis method and derivations [7–9], have concentrated on reconstructing only the dorsal portion of the SLIL; thus, volar opening and sagittal plan rotation remains a potential complication, leading to altered kinematics [3, 5].

More recently, techniques including Corella [5] and scapholunate axis (SLAM) [3] involving either a multiplanar scaphoid-lunate tether or volar reconstruction in addition to dorsal have been proposed in order to overcome this. Although preliminary studies suggest that multiple junction point techniques are better able to correct SL gap and angle compared to conventional techniques, further data, analysis and long-term follow-up studies are required to confirm this [3, 10, 11].

In this study, the finite element method together with in vitro cadaveric tests were used to investigate the performance of the modified Brunelli tenodesis (MBT), Corella and SLAM reconstruction methods in regard to their ability to restore wrist stability [6] following a simulated complete tear of the SLIL. A total of 30 3-D finite element models were created for the investigation. Neutral and ulnar-deviated clenched fist wrist positions were used to validate the models. In the latter position, virtual surgery of the MBT SLAM and Corella was performed in addition to the SLIL sectioning and non-sectioning (intact ligament) scenarios. For the neutral position, the intact (ligament) was only considered. The validation of the models was carried out through a comparison of the predicted SL dorsal gap and angle against the results obtained from the in vitro cadaveric tests.

Once the models had been validated, an investigation of the performance of the three reconstruction methods (MBT, SLAM and Corella), the intact (ligament) and the SLIL sectioning cases was undertaken with the wrist positioned at 20° flexion and 20° extension, at 15° ulnar deviation and 15° radial deviation. The predicted values of SL angle and SL gap at both dorsal and volar sides obtained from these models were used for comparison purposes between the reconstruction techniques.

The finite element method is widely employed for undertaking analyses in biomechanics offering a number of well-documented advantages compared to cadaveric studies including repeatability of analyses, ease of study parameter modification and lack of associated ethical issues. A particular advantage in utilising the finite element method in our study was that it facilitated calculation and comparison of both dorsal and volar angles for all wrist positions analysed, which is not currently feasible with the radiograph-based techniques currently employed for cadaveric/clinical studies.

## Materials and methods

### FE modelling

3-D finite element (FE) models of the right wrist from a 63-year-old female were created using 133 slices of computer tomography (CT) scan data. The slices were of 0.7 mm thickness and the transverse resolution was 512 × 512; the pixel size was 0.289 × 0.289 mm. 3-D image data processing software was used to create the surfaces of the geometry from the CT scan data. In addition, to optimise the element size of the mesh, data resampling using a pixel spacing of 0.4 × 0.4 × 0.4 mm was performed.

Reconstruction of each of the 15 cortical bone surfaces considered in the 3-D FE models was performed using a thresholding method. Masks created in the image data processing software to describe the cortical bone were exported as 3-D geometries and imported into Abaqus 6.14 (Dassault Systemes, RI, USA). The metacarpal bones were fixed and only a cortical shell was included as these geometries do not come into contact with the scaphoid and lunate bones, the main bones of interest in this study. For the remaining bones, once the cortical layer was defined, the internal volume was filled virtually and assigned trabecular bone material properties. The cortical layer thickness was determined from the CT scan and varied for each bone; the average thickness for all the bones was 2.35 mm. Metacarpal bones have an average thickness of 2.4 mm, radius and ulna 3.0 mm and the carpal bones 1.65 mm [12].

**Fig. 1:**
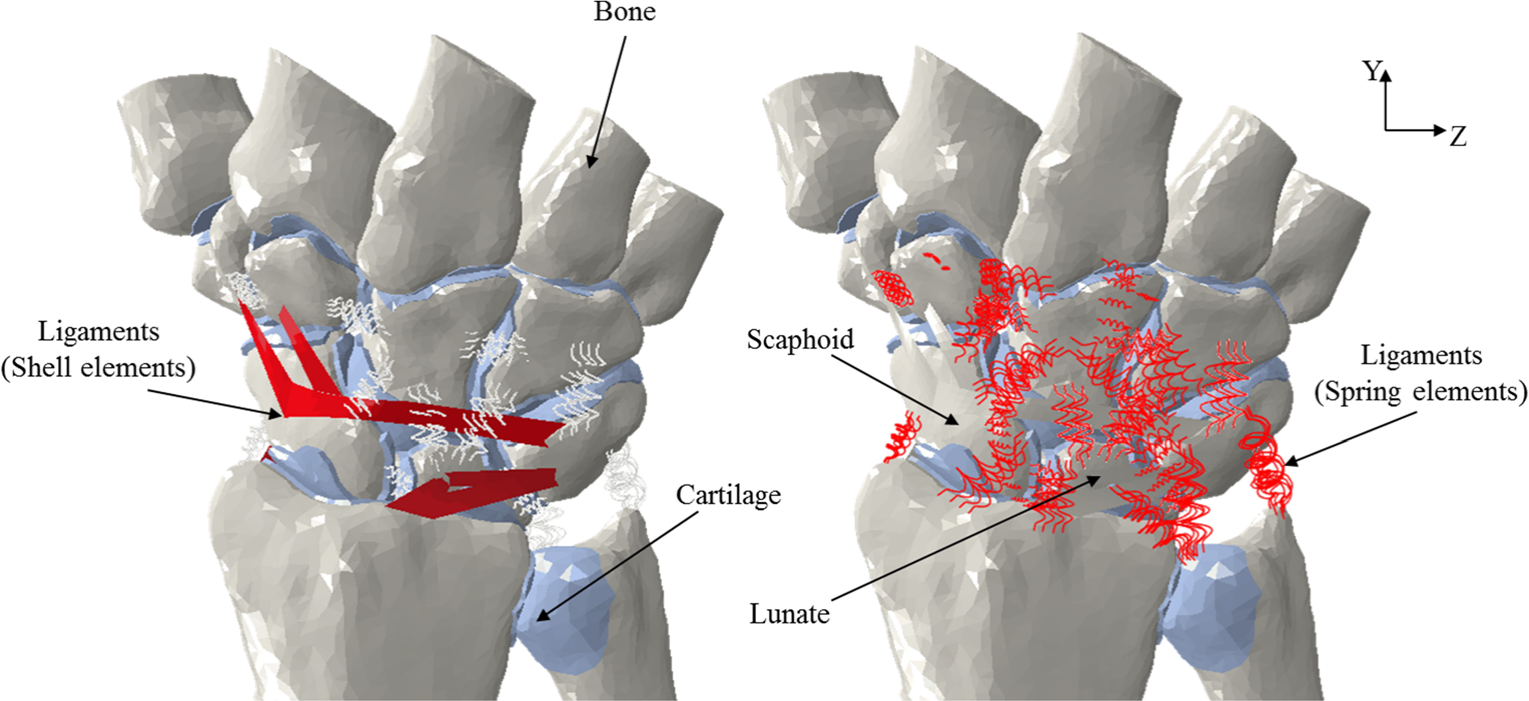
Finite element model of the wrist joint (dorsal view) showing the ligament representations

The Abaqus 6.14 software was used to create the solid mesh from the surfaces of the cortical bone geometry imported previously. Linear tetrahedral elements (C3D4) were used to mesh the bone geometry. Linear tetrahedral elements were considered adequate for the analysis in this case as the study was primarily concerned with predicting the relative position of the bones as a result of various movements rather than obtaining accurate stress estimates. The same software was used to assemble the meshed bone geometry in order to create the wrist model. Wedge elements (C3D6) were used to simulate the cartilage for the articulation between bones. The thickness of the cartilage was obtained by taking half of the distance between two articulating bones [12, 13]. Two-node spring elements defined in tension were used to simulate the 31 ligaments used in the models, 10 extrinsic ligaments, 16 intrinsic ligaments and 5 interosseous. Each of these ligaments was incorporated in the models using multiple elements allowing the distribution of the force over the area of attachment so as to avoid stress concentrations. Stiffness values of between 10 and 325 N/mm were identified from the existing literature [13–15] and applied in the FE models to the two-node spring element representations employed for the ligaments. In addition, the dorsal radiocarpal ligament, dorsal intercarpal ligament and volar radioscaphocapitate ligament, ligaments that wrap around the bone structure of the wrist anatomy, were simulated using shell elements (Fig. 1). This was achieved by first creating virtual points in the assembly following the curvature of the ligaments. The points were connected with wires to form a closed loop from which an internal surface was created which was then defined as shell geometry. Anatomic literature was used to identify the insertion points of these ligaments in the models [16, 17]. The stress-strain relationships and corresponding cross-sectional areas [17] were obtained based on the stiffness. Table 1 shows the number of elements and nodes used in each model. The neutral position of the wrist was used to set the coordinate system. For cortical and trabecular bone, a Young’s modulus of 18,000 and 100 MPa, respectively [12, 13, 19], was employed. Furthermore, a Poisson’s ratio of 0.2 was used for the cortical bone and a corresponding value of 0.25 for the trabecular bone [12, 13, 19]. Homogeneous and isotropic material behaviour was considered for the bones. Mooney-Rivlin parameters C10 of 4.1 and C01 of 0.41 were used to define the hyper-elastic material behaviour of the cartilage [12, 13]. A low value for the coefficient of friction (0.02) was assigned to the articulating surfaces in order to approximate frictionless contact [20]. Surface-to-surface contacts were assigned in the FE models to the articulations involving proximal carpal bones in order to allow free movement of triquetrum, scaphoid and lunate. In terms of the distal carpal bones, as the motion between these can be considered to be negligible [21], the articulations were assigned using tie constraints. Table 2 lists the interactions between the articulations.

**Table 1:** Ligaments included in the model

Ligament	Stiffness (N/mm)
Dorsal radiocarpal	27 [13]
Radial collateral	10 [13]
Ulnar collateral	100 [13]
Radioulnar	50 [13]
Radioscaphocapitate	50 [13]
Long radiolunate	75 [13]
Short radiolunate	75 [13]
Ulnolunate	40 [13]
Ulnotriquetral	40 [13]
Radioulnar	50 [13]
Dorsal intercarpal	128 [13]
Dorsal trapeziotrapezoid	110 [13]
Dorsal capitotrapezoid	300 [13]
Dorsal capitohamate	325 [13]
Dorsal triquetrohamate	300 [13]
Dorsal lunate-capitate	150 [13]
Dorsal lunate-hamate	150 [13]
Dorsal scaphocapitate	150 [13]
Volar trapeziotrapezoid	110 [14]
Volar scaphotrapezial	150 [13]
Volar scaphotrapezoidal	150 [13]
Volar scaphocapitate	40 [13]
Volar capitotrapezoid	80 [14]
Volar capitohamate	210 [14]
Volar triquetrocapitate	40 [13]
Volar triquetrohamate	300 [13]
Scapholunate dorsal	60 [15, 18]
Scapholunate volar	30 [15, 18]
Scapholunate proximal	15 [15, 18]
Lunotriquetral volar	250 [14]
Trapeziotrapezoid	110 [14]
Capitotrapezoid	300 [13]
Capitohamate	325 [13]

**Table 2:** Model articulation interaction

Articulations	Interaction
Lunate-capitate	Contact
Lunate-radius	Contact
Lunate-scaphoid	Contact
Lunate-triquetrum	Contact
Scaphoid-capitate	Contact
Scaphoid-radius	Contact
Scaphoid-trapezoid	Contact
Scaphoid-trapezium	Contact
Triquetrum-hamate	Contact
Triquetrum-pisiform	Tie
Radius-ulna	Contact
Capitate-metacarpals	Tie
Capitate-hamate	Tie
Capitate-trapezoid	Tie
Hamate-metacarpals	Tie
Trapezoid-trapezium	Tie
Trapezoid-metacarpals	Tie
Trapezium-metacarpals	Tie

**Fig. 2:**
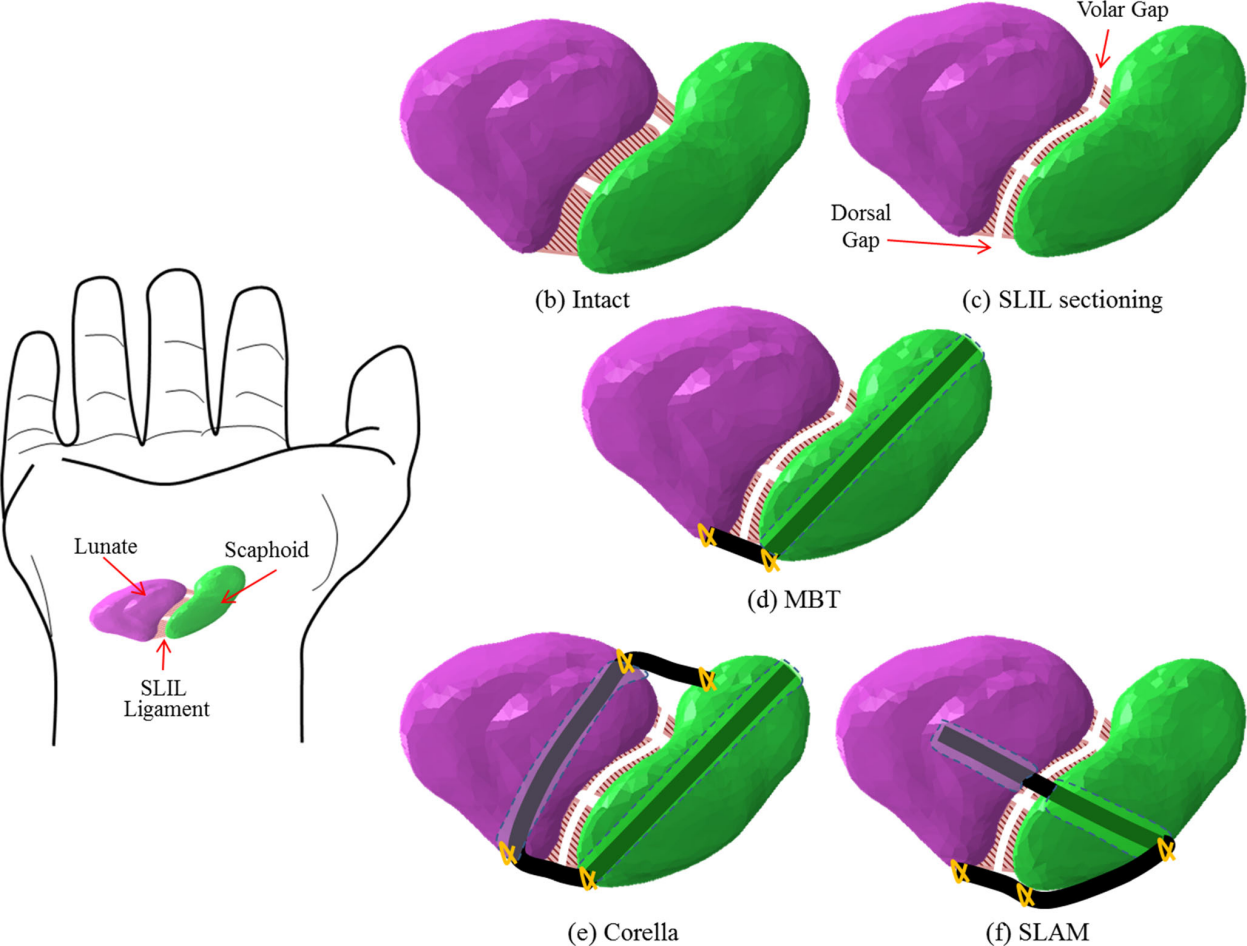
Model scenarios. **a** Hand showing lunate, scaphoid and SLIL. **b** Intact wrist. **c** SLIL sectioning. **d** Modified Brunelli tenodesis (MBT). **e** Corella. **f** SLAM model

The FE models were created based on the intact (ligament) wrist model. The models simulate three reconstruction techniques; the scapholunate axis method (SLAM), Corella and MBT, with the hand in the neutral position, in the ulnar-deviated clenched fist position, at 20° flexion, 20° extension, 15° radial deviation and 15° ulnar deviation. In addition, for each wrist position, models were created to simulate the wrist joint following SLIL sectioning, where the connection between the scaphoid and lunate bone is severed (Fig. 2c).

Virtual surgery was undertaken based on the description of the three tendon graft reconstruction techniques. In the case of the Corella reconstruction method [5], two holes are drilled one in the scaphoid and another in the lunate. The hole in the lunate bone is drilled from dorsal to volar at the medial area (Fig. 2e). The two bones (scaphoid and lunate) are then connected by tendon grafts one in the dorsal and the other in the volar region [5]. In order to perform the virtual surgery simulating the SLAM [3], the wrist is viewed in the coronal plane (Fig. 2f) and holes drilled, in the scaphoid and lunate, with tendon grafts at the dorsal and central areas used to connect the two bones. In the MBT technique [8], the tendon graft connects the lunate and scaphoid bone at the dorsal area only (Fig. 2d). This technique involves the drilling of a hole through the scaphoid bone from palmar tuberosity to a dorsal point of insertion of the dorsal SLIL. The tendon graft is then passed through the hole from volar to dorsal side.

In the FE models of the surgery, solid elements were used to mesh the 3-mm diameter cylinders shapes used to represent the tendon grafts for the three reconstruction techniques.

The tendon graft representations were assumed to be in perfect contact (tie) with the internal surfaces of the scaphoid/lunate. The cylindrical hole drilled through the bone was included in the model in order to give a more accurate representation of the surgical procedures. For the intact model, in all positions, the scaphoid and lunate were joined with SLIL Fig. 2b. The SLIL stiffness was used to represent the stiffness of the tendons grafts in the FE numerical models. An explicit algorithm was employed for FE model solution.

### Validation of the models: cadaveric study

The finite element wrist models were validated by comparing model predictions with data from a cadaveric study. Six scenarios were considered for the validation:

1. Hand in the neutral position: SLIL ligament intact
2. Ulnar-deviated clenched fist position: SLIL ligament intact
3. Ulnar-deviated clenched fist position: SLIL sectioned
4. Ulnar-deviated clenched fist position: wrist following MBT tendon graft surgery
5. Ulnar-deviated clenched fist position: wrist following SLAM tendon graft surgery
6. Ulnar-deviated clenched fist position: wrist following Corella tendon graft surgery

Fifteen cadaveric hands and wrists from specimens with a mean age of 75 years, range 54 to 94, were used for this study. The specimens were sectioned at the mid-forearm. To measure SL (dorsal) gap and angle of the specimens [22, 23], the hands were positioned in the neutral position and posteroanterior (SL) and lateral (angle) plain radiographs taken. A Steinman pin of known dimension was included in all lateral (angle) plain radiographs enabling distance to be measured accurately using calibrated software. SL gap measurement was undertaken using the methodology described by Lee et al. [23].

**Fig. 3:**
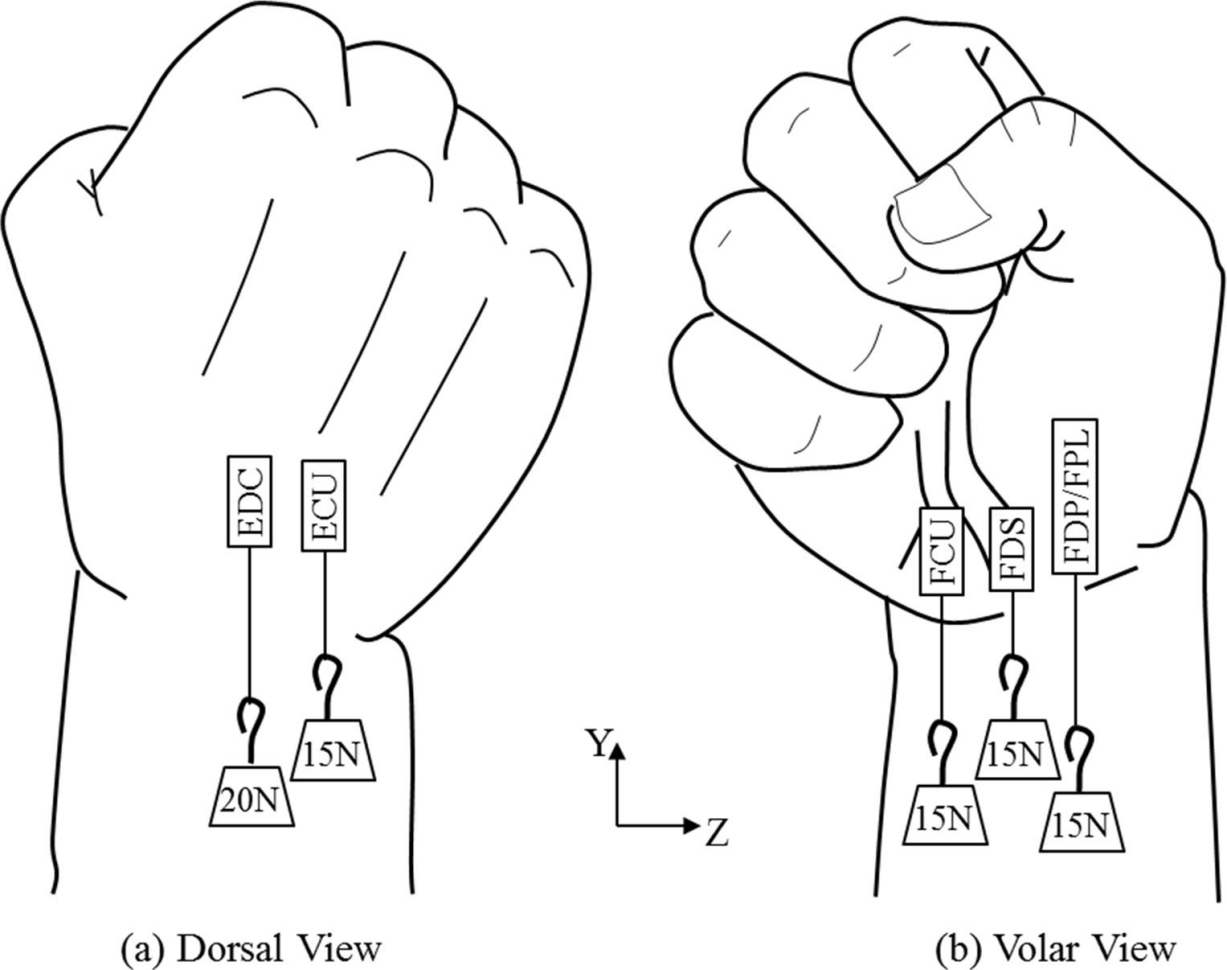
Tendon loading employed in the cadaveric study wrist model to produce the ulnar-deviated clenched fist posture. **a** Dorsal view. **b** Volar view

To be able to reproduce the motion considered in this study, six tendons of the cadaver wrists were exposed: flexor carpi ulnaris (FCU), flexor digitorum superficialis (FDS), flexor digitorum profundus (FDP), flexor pollicis longus tendon (FPL), extensor carpi ulnaris (ECU) and extensor digitorum communis (EDC). Five tendon groups (FCU, ECU, FDS, FDP/FPL and EDC) were created using FiberWire locking stitches. The ulnar-deviated clenched fist position was created by first cementing the cadaveric hands in a cylindrical plastic container which was then fixed on the edge of a table utilising a clamp. Loads of 15 and 20 N were hung separately from FDP/FPL, FCU, FDS, ECU and EDS tendons (Fig. 3).

The SLIL of all the cadaveric hands was sectioned using a scalpel blade in order to reproduce the scapholunate instability. For the position of the ulnar-deviated clenched fist, the angle and SL (dorsal) gap were determined before and after sectioning through the PA and lateral plain radiograph stress views [22, 23]. Specimens were then allocated to either the MBT, SLAM or Corella tendon graft reconstruction techniques randomly. Following the procedure, the wrists were loaded as described previously to produce the ulnar-deviated clenched fist position. Prior to and following loading, plain radiographs were taken enabling SL angle and (dorsal) gap to be ascertained.

### Cadaveric study: FE simulation

**Fig. 4:**
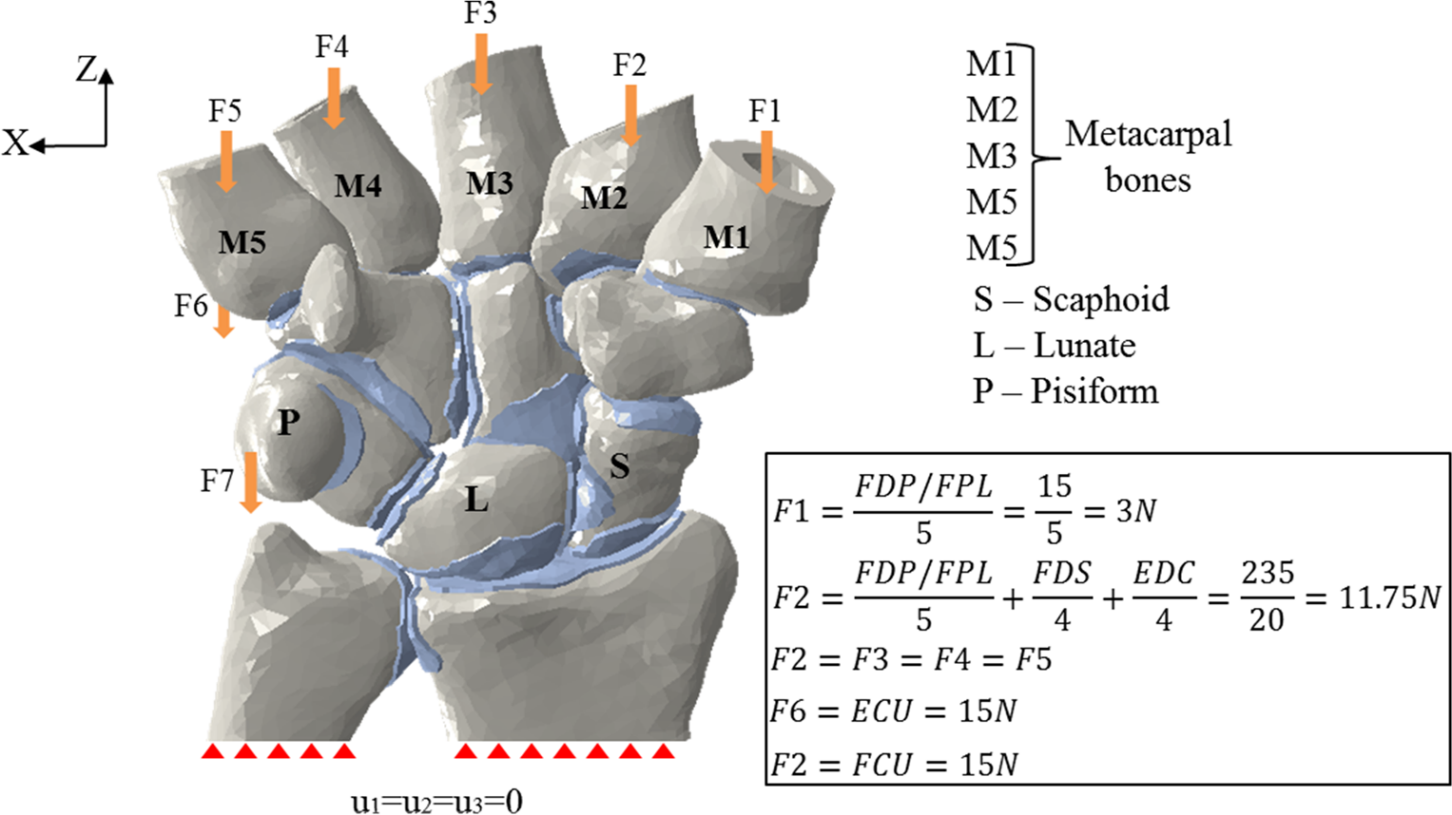
Magnitude of loads applied in the FE analysis to produce the ulnar-deviated clenched fist posture

**Fig. 5:**
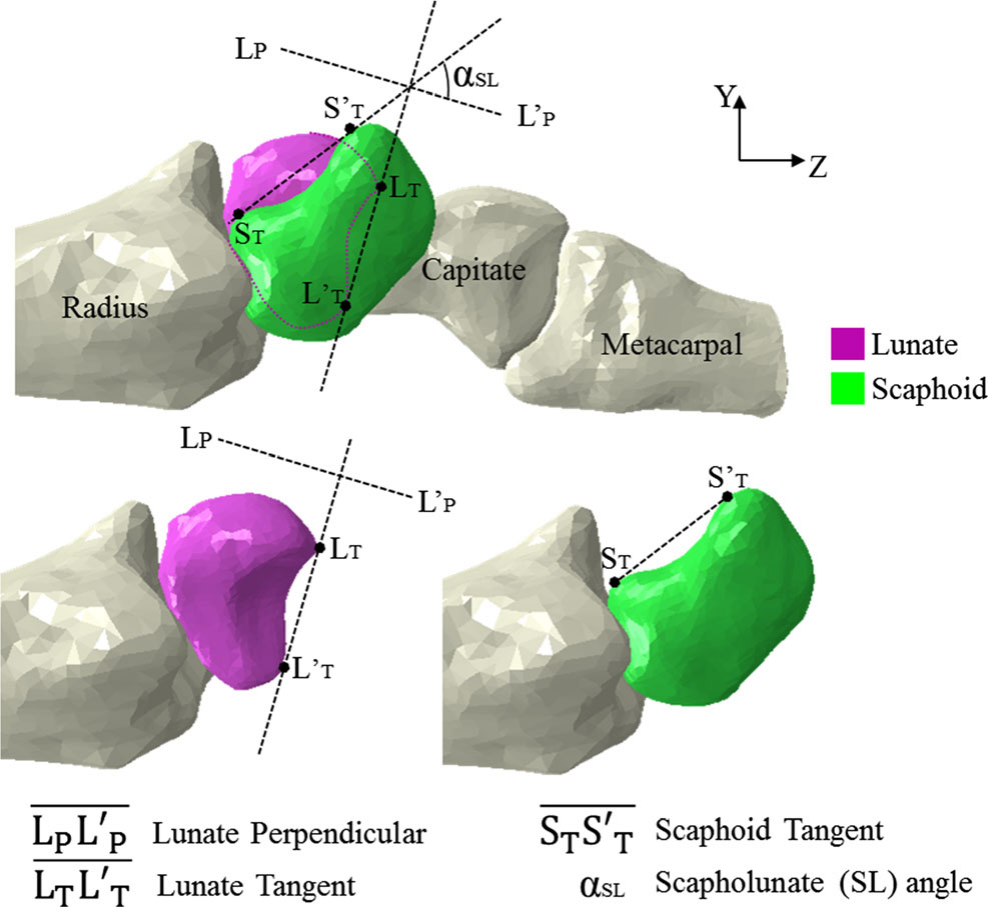
SL gap and angle calculation. SL angle from the FE model calculated using two lines in the Y-Z plane (lateral view). Line 1 (L′_P_–L_P_): perpendicular to the tangent of the two distal poles of lunate (LTLT′). Line 2 (S′–S′_T_): tangential to the palmar proximal and distal convexities of scaphoid. The SL angle is the angle between Line 1 (L′_P_–L_P_) and Line 2 (S′–S′_T_)

The experimental setup described in the above section was simulated in the model by setting a boundary condition that constrained motion in all directions at the proximal end of the ulna and radius bones (Fig. 4). The Z-axis at the proximal area of pisiform and dorsal base of the fifth metacarpal was used to apply vertical loads of 15 N in each area; hence, simulation of the loading of FCU and ECU tendons was achieved (Fig. 4). The simulation of the clenched fist position was achieved by applying a 20 N load on the EDC tendon and a 15N load on the FDS tendon, distributed equally on the medial four metacarpal bones, and a 15N load on the FDP/FPL tendon group was distributed on all the five metacarpal bones (Fig. 4). Figure 4 shows the magnitudes of the forces applied in the model. The three virtual reconstruction methods MBT, SLAM and Corella were performed with the hand in neutral position before boundary and loading conditions were applied to produce the ulna-deviated clenched fist posture, ulnar and radial deviation, and flexion and extension positions. Free motion was allowed at the lunotriquetral, scaphoid-lunate, scaphoid-capitate, scaphoid-radius, scaphoid-trapezoid, scaphoid-trapezium, lunate-capitate, lunate-radius, triquetrum-hamate and radius-ulna joints. The volar and dorsal sides SL gaps were calculated by determining the distance between the midpoints of the scaphoid and lunate articulation surface margins (Fig. 5). The method described by Larsen et al. [22] was used to calculate the SL angle (*α*) (Fig. 5). These values, SL dorsal gap and angle of each model, were used to validate the models by comparing them against the results obtained from the cadaveric study described in Section 3.1.

## Results

### FE model validation

Initial model validation consisted of comparing joint contact area and contact pressure predictions from our intact (ligament) wrist model with those determined experimentally in the cadaveric study undertaken by Tencer et al. [24]. The specimen mounting and loading conditions employed in the cadaveric study were simulated in our intact (ligament) model.

Our model predicted a contact area/total area ratio of 0.182 compared to 0.206 ± 0.0495 in the cadaveric study and a scaphoid/lunate contact area ratio of 3.02 compared to 3.72. The peak joint contact pressure predicted by the model, 4.52 MPa, was in the range determined in the cadaveric study, 2–5.6 MPa.

A mesh sensitivity analysis was undertaken to investigate model convergence. Mesh density was increased in the loaded intact ligament model until SL gap and angle changed by less than 1%. This density was then employed for subsequent analyses.

**Fig. 6:**
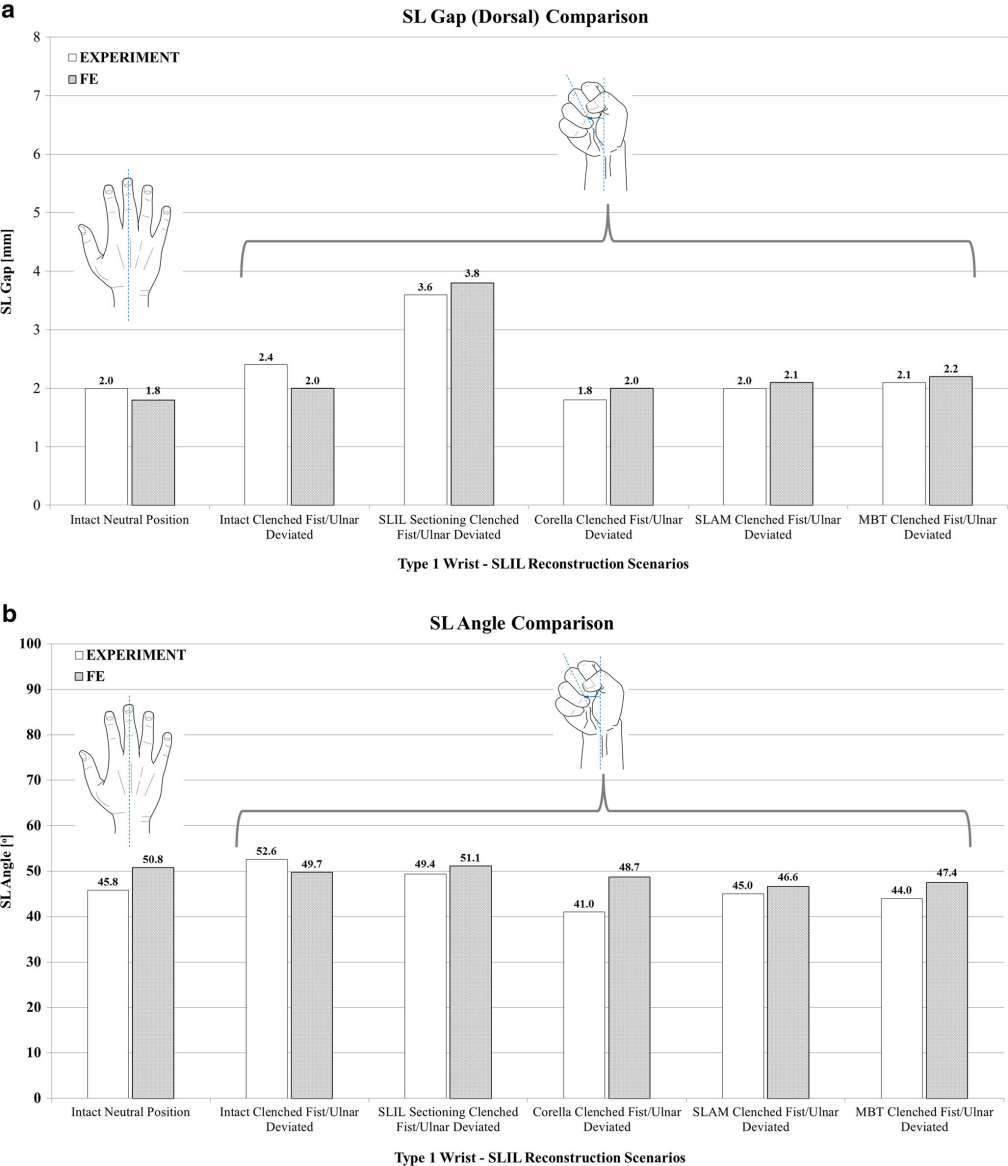
Comparison of predicted SL gap (dorsal) and angle for the six validation scenarios against the in vitro cadaveric study data. **a** SL gap. **b** SL angle

The results of the extended model validation exercise are shown in Fig. 6 where SL gap (dorsal) and SL angle predicted by the FE model for the six validation scenarios are compared against the corresponding mean values obtained from the cadaveric study. Upon inspection of the SL gap comparison shown in Fig. 6a, it can be seen that model predictions are in good agreement with the experimentally determined values; for all scenarios, the predicted SL gap is within 0.2 mm or 11% of the corresponding mean cadaveric study value except for the intact ligament in the ulna-deviated clench fist posture, where the model prediction is 0.4 mm or 16% lower than the experimental value.

A comparison of experimentally determined and FE model-predicted SL angle is presented in Fig. 6b. Generally, there is good agreement between mean SL angle obtained from the cadaver study and that predicted by our numerical model. Model predictions are within 5° and 11% of the experimental data for all scenarios except the Corella tendon reconstruction case, where the FE model predicts an SL angle which is 7.7° greater than the experimental value; however, the FE-predicted SL angle is still within the range of the cadaveric study data values, 36–53° for this scenario.

It is interesting to note that following SLIL sectioning, the SL gap increased significantly under loading compared to the intact loaded ligament case, by 1.2 mm (50%) in the experimental study and by 1.8 mm (90%) in our numerical model. In contrast, SLIL sectioning had less effect on SL angle, resulting in a mean reduction of just 3.2° (6%) in the cadaveric case and an increase of 1.4° (2.8%) in the FE model compared to the intact loaded ligament case. Tendon reconstruction reduced (dorsal) SL gap back to the original intact loaded ligament values or below in the cadaver experiments and to within 10% for our FE model cases. In addition, tendon reconstruction resulted in an SL angle less than the corresponding intact loaded ligament value in both the cadaver and FE model studies.

### Ligament reconstruction technique performance

#### Ulnar-deviated clench fist analysis

**Fig. 7:**
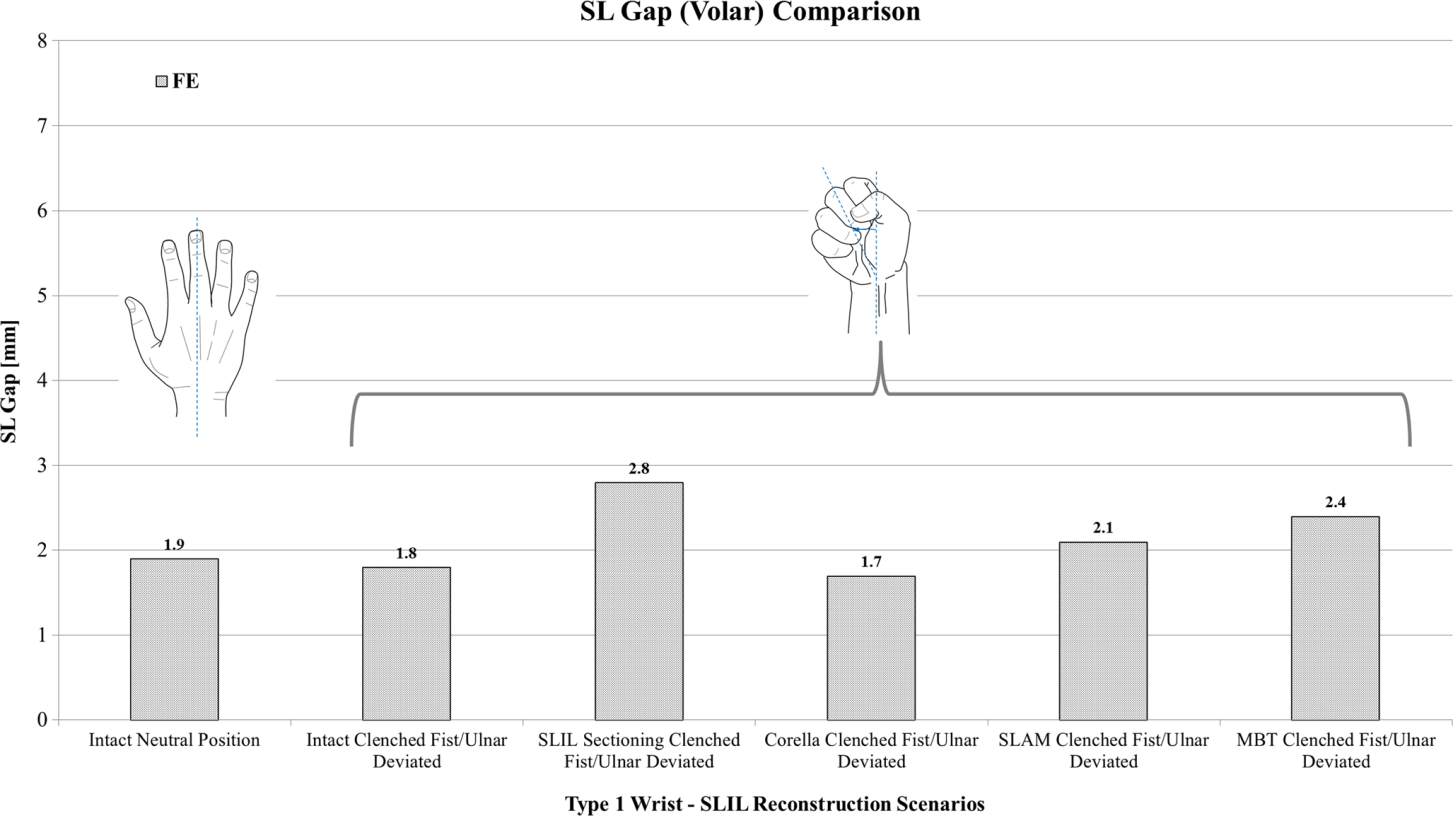
Comparison of volar SL gap predicted by the FE models for the intact ligament, SLIL-sectioned case and the MBT, SLAM and Corella ligament reconstruction methods with the wrist positioned in the ulnar-deviated clenched fist position

Figure 7 shows a comparison of volar SL gap predicted by the FE models for the intact ligament, SLIL-sectioned case and the MBT, SLAM and Corella ligament reconstruction methods with the wrist positioned in the ulnar-deviated clenched fist position. It can be seen upon inspection of this figure that sectioning caused volar SL gap to increase by almost 50% in comparison to the intact ligament case for the clenched fist posture.

Following application of the three reconstruction techniques, SL gap was restored closer to that of the intact ligament, to within 10.5% for Corella and SLAM and 26% for MBT. In terms of dorsal SL gap for the clenched fist posture (Fig. 6a), following SLIL sectioning, Corella restored SL gap back to the intact ligament value, SLAM to within 5% and MBT to within 10%. Overall, of the three reconstruction techniques, the Corella method was able to restore SL gap closer to the intact value.

#### Flexion and extension analysis

**Fig. 8:**
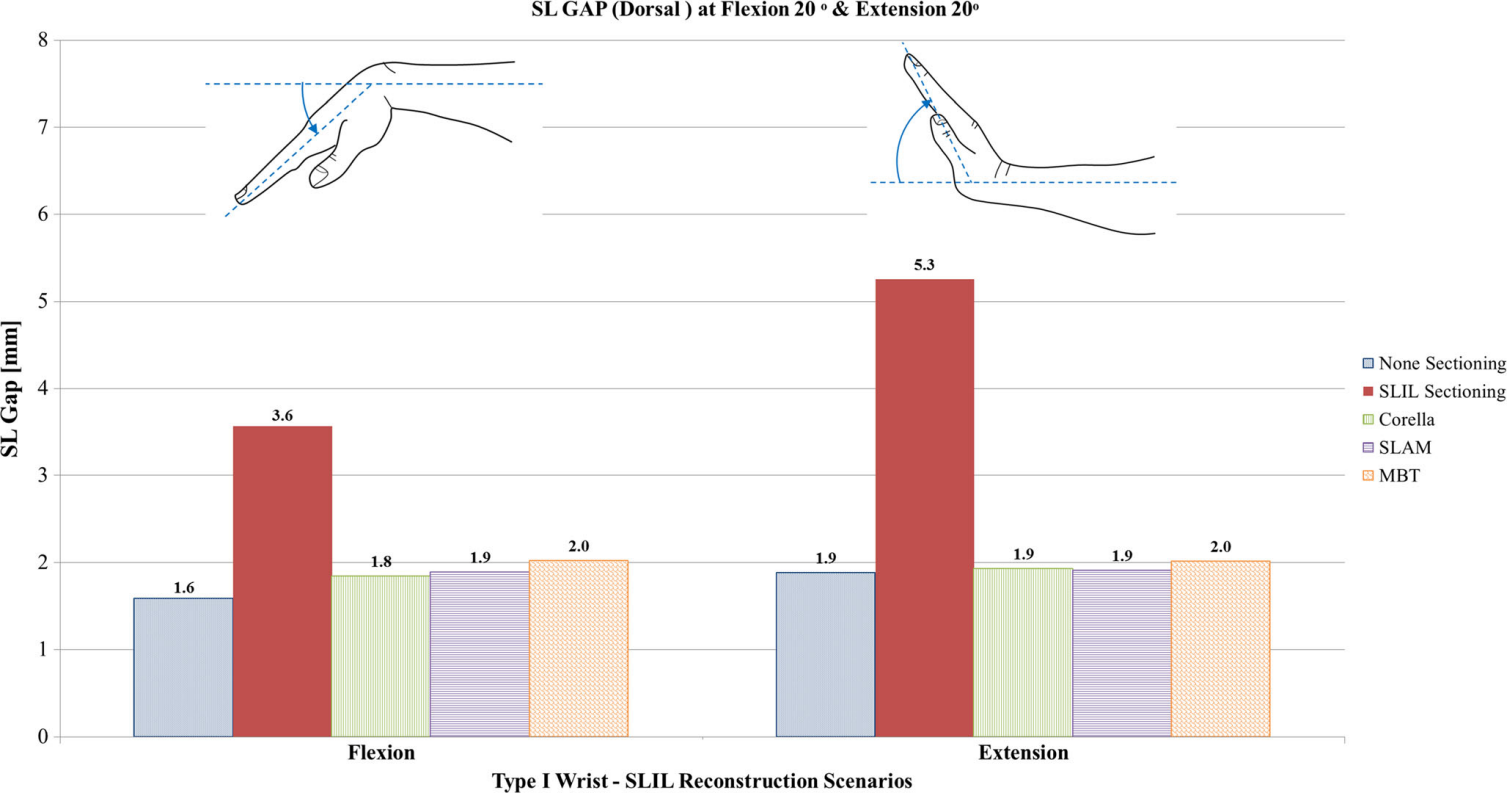
Comparison of dorsal SL gap predicted by the FE models for the intact ligament, SLIL-sectioned case and the MBT, SLAM and Corella ligament reconstruction methods with the wrist positioned at 20° flexion and 20° extension

**Fig. 9:**
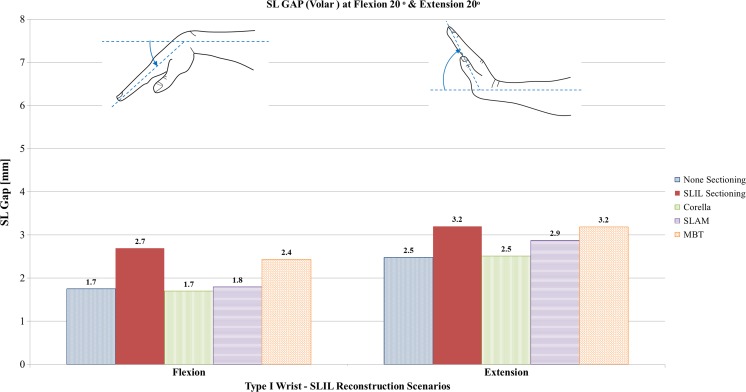
Comparison of volar SL gap predicted by the FE models for the intact ligament, SLIL-sectioned case and the MBT, SLAM and Corella ligament reconstruction methods with the wrist positioned at 20° flexion and 20° extension

**Fig. 10:**
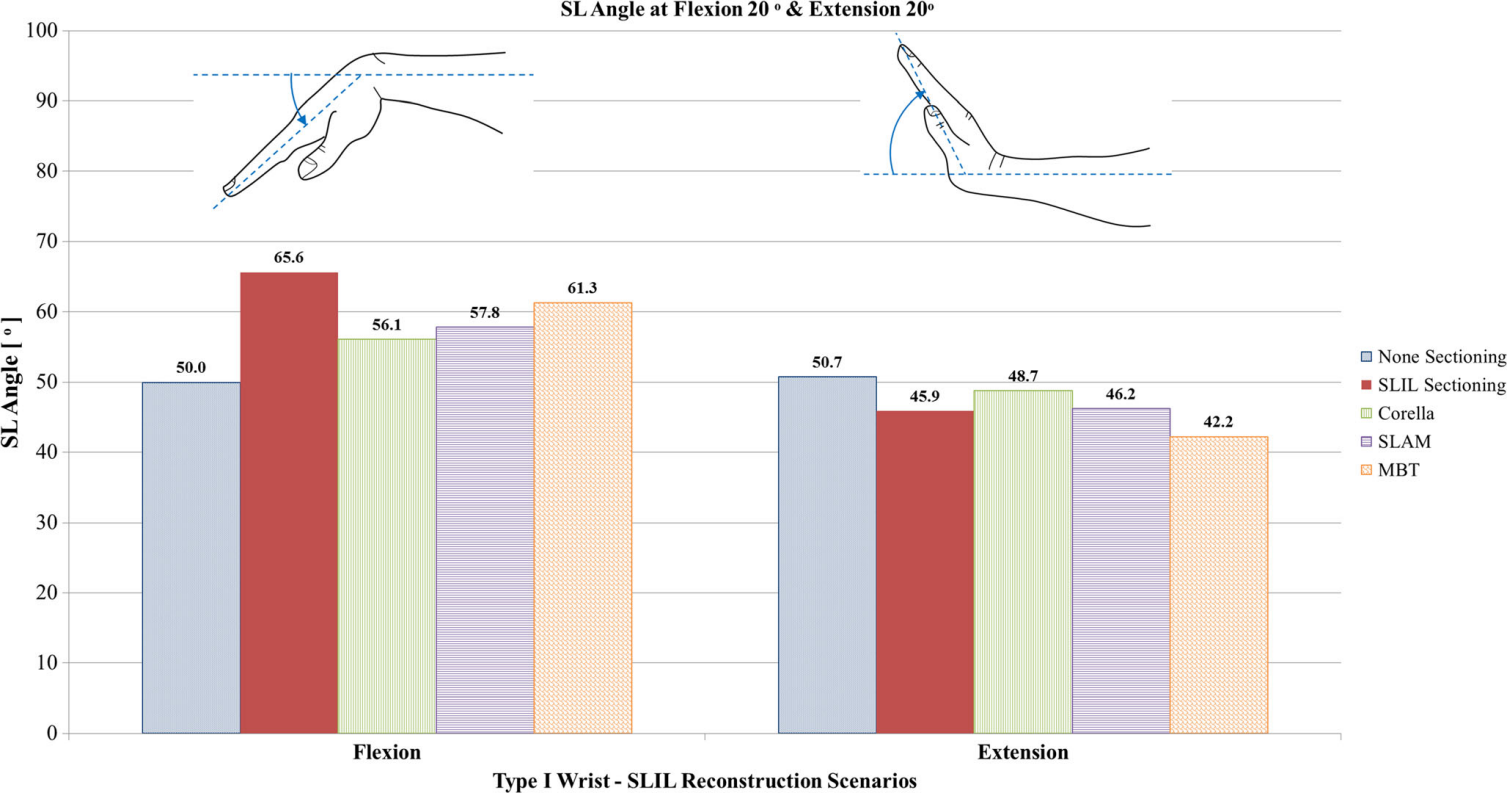
Comparison of SL angle predicted by the FE models for the intact ligament, SLIL-sectioned case and the MBT, SLAM and Corella ligament reconstruction methods with the wrist positioned at 20° flexion and 20° extension

Figures 8, 9 and 10 show a comparison of dorsal SL gap, volar SL gap and SL angle predicted by the FE models for the intact ligament, SLIL-sectioned case and the MBT, SLAM and Corella ligament reconstruction methods with the wrist positioned at 20° flexion and 20° extension.

Upon inspection of Figs. 8, 9 and 10, it can be seen that severing of the ligamentous connection between the scaphoid and lunate bone (SLIL sectioning) resulted in an increase in dorsal SL gap, by 125% in flexion and 179% in extension, compared to the intact ligament case. Volar SL gap also increased compared to the intact ligament case albeit less significantly, by 59% in flexion and 28% in extension. For the SLIL-sectioning case, SL angle was 31% greater in flexion and 10% lower in extension compared to the intact.

Of the three reconstruction techniques, the Corella method resulted in a dorsal and volar SL gap and SL angle closer to that of the intact for both flexion and extension wrist positions. The Corella technique restored volar SL gap to the same value as the intact ligament in flexion and extension, dorsal SL gap to within 12.5% and SL angle to within 12% of the intact ligament case values.

Of the SLAM and MBT methods, SLAM was better able to restore dorsal and volar SL gap and SL angle, restoring SL gap to within 19% and SL angle to within 16% of the intact for flexion and extension, compared to within 42% and 23%, respectively, for MBT. Dorsal SL gap varied least amongst the reconstruction techniques in extension; in this case, all three techniques were able to restore dorsal SL gap to within 0.1 mm (5%) or less of the intact. It is also worth noting that in extension, the MBT method resulted in the same size of volar SL gap as that obtained for the SLIL-sectioning case, 3.2 mm or 28% greater than that of the intact. SL angles were greater than the intact for the three reconstruction techniques and the SLIL-sectioning case in flexion whereas the converse was true in extension, where the SL angle was always lower than for the intact ligament.

#### Radial and ulnar deviation analysis

**Fig. 11:**
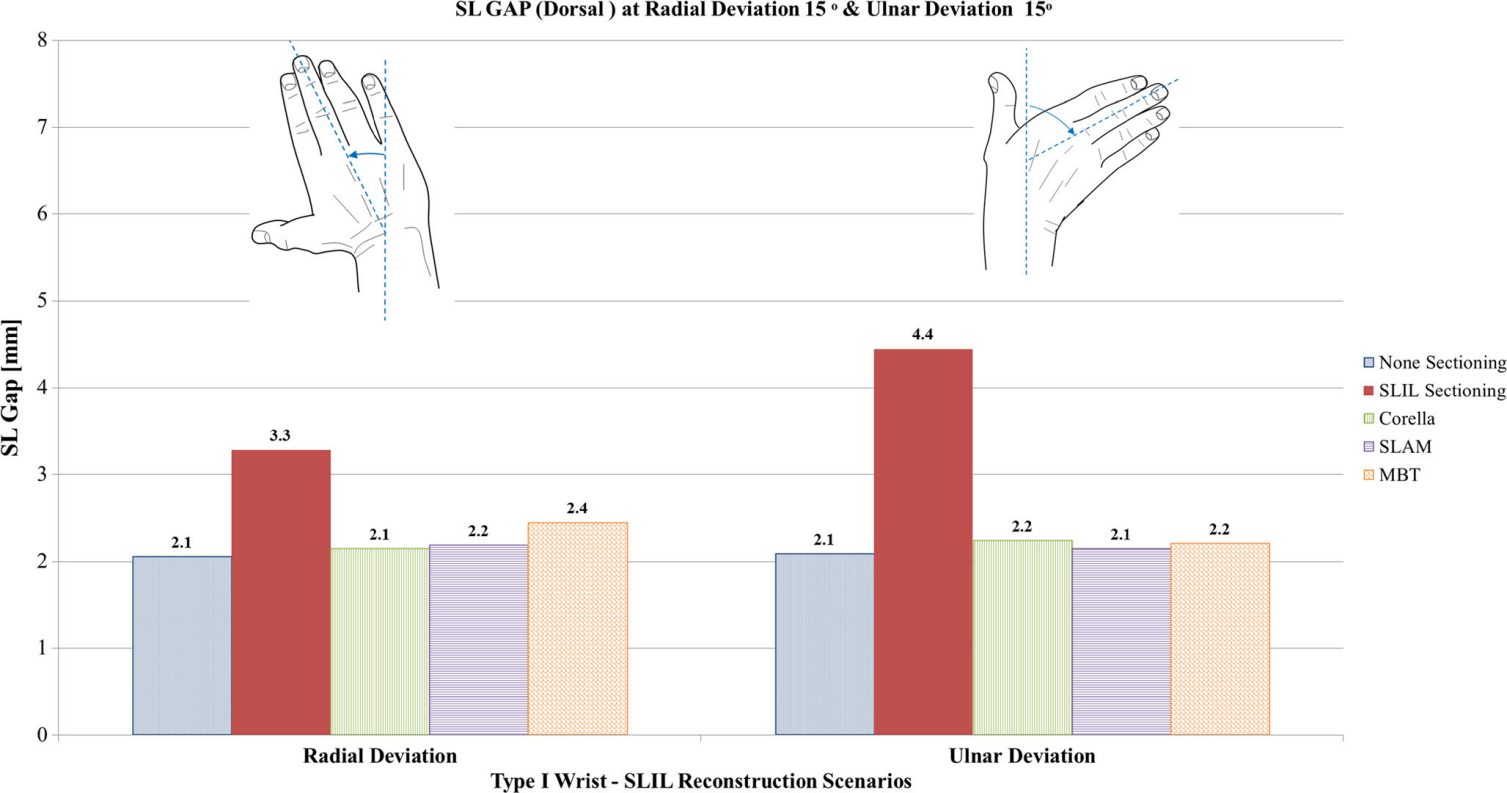
Comparison of dorsal SL gap predicted by the FE models for the intact ligament, SLIL-sectioned case and the MBT, SLAM and Corella ligament reconstruction methods with the wrist positioned at 15° radial deviation and 15° ulnar deviation

**Fig. 12:**
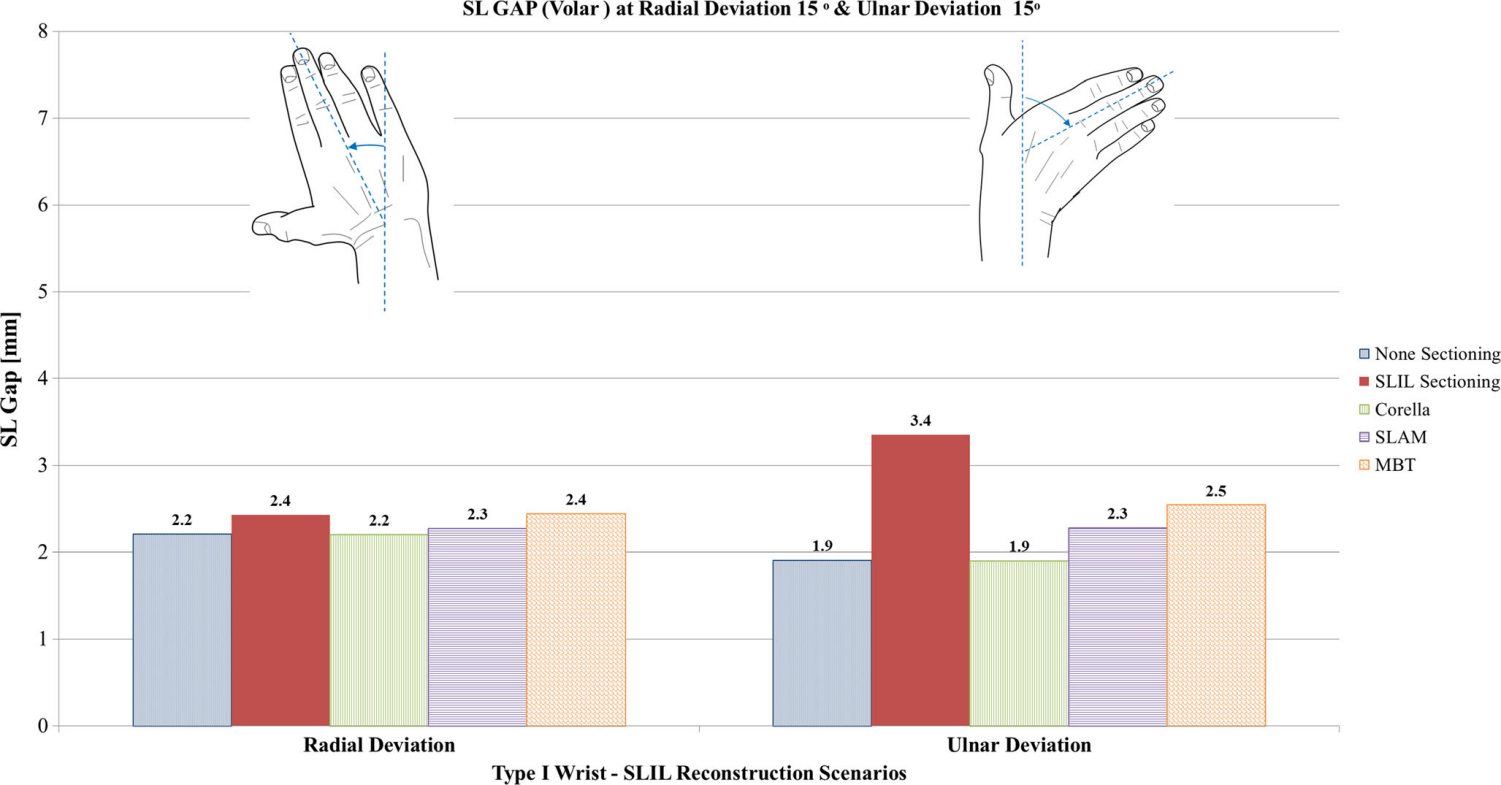
Comparison of volar SL gap predicted by the FE models for the intact ligament, SLIL-sectioned case and the MBT, SLAM and Corella ligament reconstruction methods with the wrist positioned at 15° radial deviation and 15° ulnar deviation

**Fig. 13:**
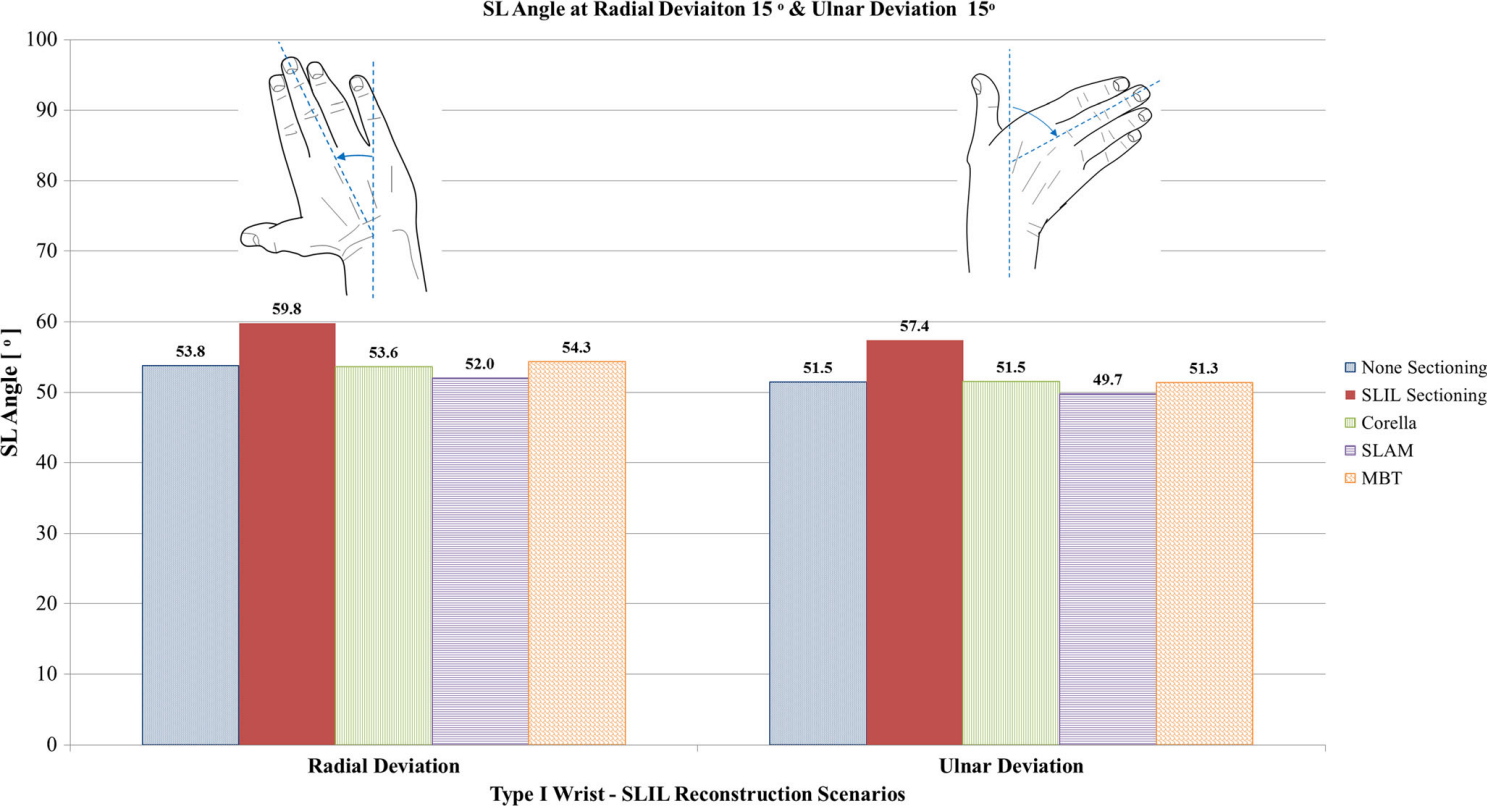
Comparison of SL angle predicted by the FE models for the intact ligament, SLIL-sectioned case and the MBT, SLAM and Corella ligament reconstruction methods with the wrist positioned at 15° radial deviation and 15° ulnar deviation

Figures 11, 12 and 13 show a comparison of dorsal SL gap, volar SL gap and SL angle predicted by the FE models for the intact ligament, SLIL-sectioned case and the MBT, SLAM and Corella ligament reconstruction methods with the wrist positioned at 15° radial deviation and 15° ulnar deviation.

Upon inspection of Figs. 11, 12 and 13, it can be seen that SLIL sectioning resulted in an increase in dorsal SL gap, by 57% in radial deviation and 110% in ulnar deviation, compared to the intact ligament case. Volar SL gap also increased compared to the intact ligament case, by 9% in radial deviation and 79% in ulnar deviation. In addition, SL angle was 11% greater in both radial and ulnar deviation after SLIL sectioning compared to the intact ligament.

Overall, of the tendon reconstruction methods simulated, the Corella method resulted in a dorsal and volar SL gap and SL angle closer to that of the intact ligament case for radial and ulnar deviation positions of the wrist.

Application of the Corella technique restored volar SL gap to that of the intact ligament (2.5 mm), dorsal SL gap to within 5% and SL angle to within 0.5% of the intact ligament values.

Of the SLAM and MBT methods, SLAM was better able to restore dorsal and volar SL gap, restoring SL gap to within 21% of the corresponding intact ligament values for radial and ulnar deviation. However, the MBT tendon reconstruction technique was able to restore SL angle closer to that of the intact, within 1%, compared to 3.5% for SLAM.

Dorsal SL gap varied least amongst the reconstruction techniques in ulnar deviation, with all three techniques able to restore dorsal SL gap to within 0.1 mm (5%) or less of the intact. Volar SL gap varied greatest amongst the reconstruction techniques in ulnar deviation; whilst the Corella technique was able to restore the volar SL gap to that of the intact ligament, the SLAM and MBT techniques produced a volar SL angle that was 21% and 32% greater than the intact case, respectively.

SL angle was equal to or lower than the intact case for the three reconstruction techniques, whereas the angle was greater for SLIL sectioning.

## Discussion

A variety of treatments currently exist for treating chronic SL instability. Ligamentous reconstruction techniques including capsulodesis, bone-ligament-bone and tenodesis are an option where patients present with non-repairable SLIL injury but a reducible SL dissociation [5]. Until recently, tenodesis procedures have concentrated on reconstructing the dorsal component of SLIL thus volar opening and sagittal plan rotation leading to altered kinematics remains a potential complication [3, 5]. More recently, techniques including Corella and SLAM involving either a multiplanar scaphoid-lunate tether or volar reconstruction in addition to dorsal have been proposed in order to overcome this.

Using a finite element model and cadaveric study data, we investigated the performance of the Corella, SLAM and MBT techniques.

SL gap and angle predictions from our model were in good agreement with those from the cadaveric study for the six scenarios considered, including the three ligament reconstruction techniques MBT, SLAM and Corella. SL gap predictions were all within 0.4 mm and SL angles within 7.7° of the corresponding experimental mean data values. The cadaveric data and predictions from our model showed that SLIL sectioning had a much greater effect on SL gap compared to angle, with experimentally obtained mean SL gap increasing by 50% or 1.2 mm compared to the intact ligament case for the ulnar-deviated clench fist position. In this case, SL gap values met the criteria for SL dissociation [4] and were in good agreement with another study that measured SL gap following ligamentous sectioning in cadaver wrists loaded to produce an ulna-deviated clench fist posture [23]. In contrast, the effect of sectioning on SL angle was relatively minor, causing a change of no more 6%, with SL angle remaining within the reportedly normal range, 30° to 60° [25]. These results concur with those of other researchers who determined that solely dividing the SLIL does not have a significant effect on the rotational motion of the scaphoid and lunate for radial-ulnar deviation [26].

For the ulnar-deviated clenched fist posture investigated, the tendon reconstruction techniques reduced dorsal SL gap to within 10% of the values obtained for the original intact loaded ligament and volar SL gap to within 26% in all cases whilst maintaining SL angle in the normal range, demonstrating the techniques’ abilities to restore dorsal SL gap following non-repairable SLIL injury. Of the three tendon reconstruction techniques, Corella was more effective in restoring SL gap, restoring dorsal gap back to the original intact loaded value and volar gap to within 10.5%.

In flexion-extension, the lunate rotates over the radius “in the dorsal direction” during flexion and “in the volar direction” during extension [6]; therefore, for any tendon reconstruction technique, reduction and stabilisation of the dorsal gap is of significant importance in flexion, whereas for extension the volar gap is particularly important. Our model demonstrated that all three reconstruction techniques, Corella, MBT and SLAM, were able to restore dorsal SL gap to within 0.4 mm of the intact ligament during flexion and 0.1 mm during extension following simulated SLIL sectioning. This is as expected, as all three techniques involve reconstruction of the dorsal portion of the SLIL. Of the three techniques, Corella was able to restore dorsal SL gap and angle closer to that of the intact ligament, followed by SLAM then MBT. However, greater variation was found between the techniques in terms of their ability to restore volar SL gap, with the techniques involving either a multiplanar scaphoid-lunate tether (SLAM) or reconstruction of the volar portion of the SLIL in addition to the dorsal (Corella), performing better. In flexion and extension, the Corella technique was able to restore volar SL gap to the same as that for the intact ligament. In flexion, SLAM restored dorsal SL gap to within 5.8% of the intact ligament, but fared less well in extension, where volar opening is more significant, only being able to restore volar SL gap to within 16% of the intact. The MBT technique, which reconstructs just the dorsal portion of the SLIL, was not able to reduce volar SL gap at all compared to the SLIL-sectioned case in extension, and only by 11% in flexion.

The predictions from our FE models indicated that for radial and ulnar deviation, all three reconstruction techniques simulated were able to restore dorsal SL gap to within 0.3 mm and SL angle to within 1.8° of the intact ligament following SLIL sectioning. Again, this is not unexpected as all techniques involve dorsal SLIL portion reconstruction. However, more variation was found in the ability of the techniques to restore dorsal SL gap. The Corella technique, which involves reconstruction of the volar portion of the SLIL, restored volar SL gap back to that of the intact ligament for both radial and ulnar deviation. SLAM, which involves a multiplanar scaphoid-lunate tether, was able to restore volar SL gap to within 4.5% of the intact ligament in radial deviation and 21% in ulnar deviation, whereas the corresponding values for MBT, which reconstructs only the dorsal portion of the SLIL, were 9% and 32%, respectively.

The relative significance of the various portions of the SLIL is still undecided, with conflicting data available in the literature [27]. However, the results from our study indicate that unless ligamentous reconstruction techniques involve multiple junction points between scaphoid and lunate, volar gap widening and sagittal plane rotation is likely to occur which may consequently lead to altered kinematics. Of the three reconstruction techniques considered, overall, we found Corella was better able to restore both dorsal and volar SL gap and SL angle following SLIL injury; however, further analysis and long-term clinical follow-up studies are required to confirm outcomes and evaluate potential creep and elongation with the reconstruction.

Limitations and assumptions apply to our study which are typical of complex numerical analyses in the field of biomechanics. A number of assumptions and simplifications were inevitably required including geometrical representations and material properties. In terms of soft tissue representations, a hyper-elastic material model was employed for cartilage which is considered to provide a more accurate representation of behaviour [12, 13].

The majority of the ligaments included in the model were represented using spring elements. Whilst the use of one-dimensional representations of ligament geometries is commonplace in biomechanical joint models and has been shown to be valuable particularly for investigating kinematics where external loading is present, a number of limitations have been identified, including the inability to accurately capture non-uniform 3-D stress and strain, non-uniform deformations and joint orientation effects [28]. Three-dimensional FE modelling approaches have been highlighted as being required for more accurate ligament representation; however, it is recognised that this is not straightforward and can be massively time-consuming [28]. Linear elastic material properties were employed for the ligaments. In reality, ligaments typically exhibit non-linear viscoelastic behaviour so if ligament strain was low, then behaviour would fall within the non-linear region and ligament stiffness would be overestimated as a result which would affect joint motion prediction. However, accurate data for the large number of parameters required to describe non-linear viscoelastic ligament behaviour is not readily available [28]. Furthermore, it has been determined that ligaments tend to operate at or close to the linear region, so an assumption of linear elastic behaviour should not introduce significant error [29].

We validated our finite element wrist model by comparing predicted SL gap and SL angle with data obtained from an in vitro cadaveric study conducted on intact, SLIL-sectioning specimens and cadaveric wrists following simulation of the three reconstruction techniques with the hand in the neutral position and under ulnar-deviated clenched fist posture. The good agreement between predicted and experimentally obtained SL gap and angle data suggests that our model is able to model and represent behaviour to a good degree of accuracy and the assumptions used in the model do not introduce significant error.

We used CT scans from the wrist of a single volunteer for creating our FE models; therefore, caution should be taken from drawing extensive conclusions from the results. That said, model predictions compared well with mean results from simulation of the three reconstruction techniques undertaken on 15 cadaveric wrists; therefore, our wrist model appears to be a good representation and a certain degree of confidence can be placed in the results from it.

Although SLIL sectioning only partly replicates scapholunate instability, it is recognised that the SLIL is the primary stabiliser of the joint [30]. To better simulate scapholunate instability, our model could be revised to take into account stretching of the supporting ligaments, in particular the radioscaphocapitate and dorsal intercarpal ligaments; however, the soft tissue reconstruction techniques we model are generally employed for early chronic or subacute SLIL disruptions before secondary constraints have been excessively compromised and irreducible SL subluxation has occurred [3].

Fixation of tendon reconstructions to bone is treated similarly in every reconstructive option we modelled [20], which may not be the clinical scenario. Our models focus on the relative position of the bones after the reconstruction scenarios. The simulations assume that the tendon graft is attached to a point which would not change if it is a suture, screw or tunnel anchor. Suture to a soft tissue could influence the results in that the tissue has some flexibility which allows relative motion of the tendon graft when it starts to deform.

SL gap and angle measurement of the cadaveric wrist specimens was based on posteroanterior (SL) and lateral (angle) plain radiographs and therefore any rotational error in X-ray positioning could potentially affect accuracy.
